# Analysis of Mortality among Neonates and Children with Spina Bifida: An International Registry‐Based Study, 2001‐2012

**DOI:** 10.1111/ppe.12589

**Published:** 2019-10-21

**Authors:** Marian K. Bakker, Vijaya Kancherla, Mark A. Canfield, Eva Bermejo‐Sanchez, Janet D. Cragan, Saeed Dastgiri, Hermien E. K. De Walle, Marcia L. Feldkamp, Boris Groisman, Miriam Gatt, Paula Hurtado‐Villa, Karin Kallen, Daniella Landau, Nathalie Lelong, Jorge S. Lopez Camelo, Laura Martínez, Margery Morgan, Osvaldo M. Mutchinick, Wendy N. Nembhard, Anna Pierini, Anke Rissmann, Antonin Sipek, Elena Szabova, Giovanna Tagliabue, Wladimir Wertelecki, Ignacio Zarante, Pierpaolo Mastroiacovo

**Affiliations:** ^1^ University of Groningen University Medical Center Groningen Department of Genetics Eurocat Northern Netherlands Groningen The Netherlands; ^2^ Department of Epidemiology Emory University Rollins School of Public Health Atlanta GA USA; ^3^ Birth Defects Epidemiology and Surveillance Branch Texas Department of State Health Services Austin TX US; ^4^ ECEMC (Spanish Collaborative Study of Congenital Malformations) CIAC Instituto de Investigación de Enfermedades Raras (IIER) Instituto de Salud Carlos III Madrid Spain; ^5^ Division of Congenital and Developmental Disorders National Center on Birth Defects and Development Disabilities Centers for Disease Control Atlanta GA USA; ^6^ Health Services Management Research Centre Tabriz University of Medical Sciences Tabriz Iran; ^7^ Department of Pediatrics University of Utah School of Medicine and the Utah Birth Defect Network Salt Lake City UT USA; ^8^ National Network of Congenital Anomalies of Argentina (RENAC) National Center of Medical Genetics National Administration of Laboratories and Health Institutes (ANLIS) National Ministry of Health Buenos Aires Argentina; ^9^ Malta Congenital Anomalies Registry Directorate for Health Information and Research Valetta Malta; ^10^ Department of Basic Sciences of Health School of Health Pontificia Universidad Javeriana Cali Cali Colombia; ^11^ National Board of Health and Welfare and University of Lund Stockholm Sweden; ^12^ Department of Neonatology Soroka Medical Center Beer‐Sheva Israel; ^13^ Inserm UMR 1153 Obstetrical Perinatal and Pediatric Epidemiology Research Team (Epopé) Center for Epidemiology and Statistics Sorbonne Paris Cité DHU Risks in Pregnancy Paris Descartes University Paris France; ^14^ ECLAMC Center for Medical Education and Clinical Research (CEMIC‐CONICET) Buenos Aires Argentina; ^15^ Genetics Department Hospital Universitario Dr Jose E. Gonzalez Universidad Autonóma de Nuevo León San Nicolás de los Garza Mexico; ^16^ CARIS, The Congenital Anomaly Register for Wales Singleton Hospital Swansea UK; ^17^ RYVEMCE Department of Genetics Instituto Nacional de Ciencias Médicas y Nutrición Salvador Zubirán Mexico City Mexico; ^18^ Department of Epidemiology, Arkansas Center for Birth Defects Research and Prevention and Arkansas Reproductive Health Monitoring System Fay Boozman College of Public Health University of Arkansas for Medical Sciences Little Rock AR USA; ^19^ Institute of Clinical Physiology National Research Council and Fondazione Toscana Gabriele Monasterio Tuscany Registry of Congenital Defects Pisa Italy; ^20^ Malformation Monitoring Centre Saxony‐Anhalt Medical Faculty Otto‐von‐Guericke University Magdeburg Germany; ^21^ Department of Medical Genetics Thomayer Hospital Prague Czech Republic; ^22^ Slovak Teratologic Information Centre (FPH) Slovak Medical University Bratislava Slovak Republic; ^23^ Lombardy Congenital Anomalies Registry Cancer Registry Unit Fondazione IRCCS Istituto Nazionale tumori Milan Italy; ^24^ Omni‐Net for Children International Charitable Fund Rivne Rivne Ukraine; ^25^ Human Genetics Institute Pontificia Universidad Javeriana Bogotá Colombia; ^26^ International Center on Birth Defects International Clearinghouse for Birth Defects Surveillance and Research Rome Italy

**Keywords:** epidemiology, mortality, registry‐based study, spina bifida

## Abstract

**Background:**

Medical advancements have resulted in better survival and life expectancy among those with spina bifida, but a significantly increased risk of perinatal and postnatal mortality for individuals with spina bifida remains.

**Objectives:**

To examine stillbirth and infant and child mortality among those affected by spina bifida using data from multiple countries.

**Methods:**

We conducted an observational study, using data from 24 population‐ and hospital‐based surveillance registries in 18 countries contributing as members of the International Clearinghouse for Birth Defects Surveillance and Research (ICBDSR). Cases of spina bifida that resulted in livebirths or stillbirths from 20 weeks' gestation or elective termination of pregnancy for fetal anomaly (ETOPFA) were included. Among liveborn spina bifida cases, we calculated mortality at different ages as number of deaths among liveborn cases divided by total number of liveborn cases with spina bifida. As a secondary outcome measure, we estimated the prevalence of spina bifida per 10 000 total births. The 95% confidence interval for the prevalence estimate was estimated using the Poisson approximation of binomial distribution.

**Results:**

Between years 2001 and 2012, the overall first‐week mortality proportion was 6.9% (95% CI 6.3, 7.7) and was lower in programmes operating in countries with policies that allowed ETOPFA compared with their counterparts (5.9% vs. 8.4%). The majority of first‐week mortality occurred on the first day of life. In programmes where information on long‐term mortality was available through linkage to administrative databases, survival at 5 years of age was 90%‐96% in Europe, and 86%‐96% in North America.

**Conclusions:**

Our multi‐country study showed a high proportion of stillbirth and infant and child deaths among those with spina bifida. Effective folic acid interventions could prevent many cases of spina bifida, thereby preventing associated childhood morbidity and mortality.

**Synopsis**

1 **Study question**

To examine perinatal and infant and child mortality and its trends for those affected with spina bifida.

2 **What is already known**

Medical advancements have resulted in better survival and life expectancy among those with spina bifida, but a significantly increased risk of perinatal and infant and child mortality remains.

3 **What this study adds**

Our multi‐country study showed perinatal and infant and child mortality is a major concern for those with spina bifida. The overall first‐week mortality proportion was lower in programmes with policies that allowed elective terminations of pregnancy for fetal anomalies compared with those that did not. The proportion of perinatal and infant and child deaths were higher among spina bifida cases with co‐occurring unrelated major anomalies or genetic syndromes compared with those with isolated spina bifida.

## 1 BACKGROUND

Spina bifida is a common and major congenital disorder of the central nervous system characterised by incomplete or incorrect closure of the neural tube during the embryonic development.^1^ Spina bifida affects over 150 000 births worldwide and contributes to significant disability and child mortality.^2^ The observed prevalence of spina bifida varies globally and is largely influenced by differences in surveillance methods, prenatal diagnosis and elective termination policies, and folic acid fortification of staple foods in a given country or region.^3^, ^4^, ^5^ Evidence from both randomised clinical trials and observational studies shows that many cases of spina bifida can be prevented by women taking 400‐800 mcg/day of folic acid during preconception and early pregnancy.^6^, ^7^, ^8^, ^9^ The United States Preventive Services Task Force (USPSTF) recommends that all women planning or capable of pregnancy take a daily supplement containing 0.4‐0.8 mg (400‐800 mcg) of folic acid.^10^

Medical advancements since the 1960s, especially in developed countries, have resulted in better survival and life expectancy among those with spina bifida.^11^ But even with improved medical care, studies show a significantly increased risk of perinatal and postnatal mortality for individuals with spina bifida compared to those without.^1^, ^12^, ^13^, ^14^, ^15^, ^16^, ^17^, ^18^ Mortality associated with spina bifida is more frequent in countries with fewer resources and less health care access compared with their counterparts in high‐income regions of the world.^1^, ^2^, ^4^, ^5^ Few studies have been conducted that examined mortality associated with spina bifida, and most of them were conducted in developed countries.^15^, ^16^, ^19^, ^20^ Wang et al conducted a population‐based analysis examining the survival of children with spina bifida in New York State in the United States. In this large population‐based study examining children born with spina bifida between years 1983 and 2006, the probability of survival was 93% up to age 7 days, 92% up to 1 month, 88% up to 1 year, 86% up to 5 years, and 82% up to 25 years.^16^ Time trends in spina bifida survival are also not well examined globally; two studies from the United States and Canada showed an improvement in survival among cohorts born in later years compared with those born during the late 1970s and the early 1980s.^17^, ^20^, ^21^

There are opportunities to study mortality among infants born with spina bifida utilising pooled data from large networks of established birth defects surveillance systems, which have a potential to link to death certificates or other administrative health data sets. The primary objective of our study was to examine perinatal and infant and child mortality for those affected by spina bifida using data from multiple birth defects registries affiliated with the International Clearinghouse for Birth Defects Surveillance and Research (ICBDSR) and examine temporal trends in mortality. As a secondary objective, we examined the total prevalence of spina bifida using data from participating programmes. We were also able to stratify by the availability of elective termination for fetal anomalies (ETOPFA) on perinatal and infant and child mortality.

## 2 METHODS

### 2.1 Study design and setting

International Clearinghouse for Birth Defects Surveillance and Research was established in 1974 and is a voluntary non‐profit organisation affiliated with the World Health Organization (http://www.icbdsr.org/). As a consortium of birth defects surveillance and research programmes from around the world, ICBDSR investigates and aims to prevent birth defects and minimise any negative consequences associated with them. As of 2018, there are 42 birth defects surveillance programmes in ICBDSR, either population‐based or hospital‐based, of which 27 contribute data on an annual basis. These registries provide aggregated data on children and fetuses affected with at least one of 39 different birth defects to ICBDSR for surveillance purposes (a list of all monitoring programmes and their surveillance attributes can be found at http://www.icbdsr.org/wp-content/annual_report/Report2014.pdf). Each programme also collects data on the total annual number of livebirths and stillbirths in their source population for each of the surveillance years to aid in prevalence estimation.

**Figure 1:**
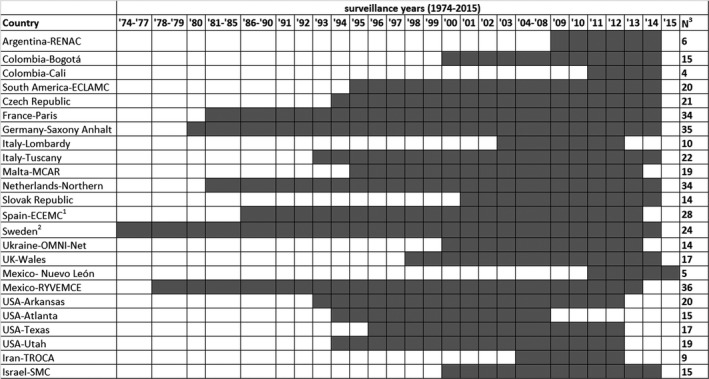
Spina bifida surveillance period by country and registry, International Clearinghouse for Birth Defects Surveillance and Research. ^1^Spain included information on elective termination of pregnancy for fetal anomalies from 1995 to 2014. ^2^Sweden included information on elective terminations of pregnancy for fetal anomalies from 1999 to 2014. ^3^Number of surveillance years. ECEMC, Registry of the Spanish Collaborative Study of Congenital Malformations; ECLAMC, Latin American Collaborative Study of Congenital Malformations; MCAR, Malta Congenital Anomalies Registry; OMNI‐Net = Ukraine Birth Defects Prevention Program; RENAC, National Network of Congenital Anomalies of Argentina; RYVEMCE, Mexican Registry and Epidemiological Surveillance of External Congenital Malformations; TROCA, Tabriz Registry of Congenital Anomalies; SMC, Soroka Medical Center; UK, United Kingdom; USA, United States of America

For the current analysis, each programme contributed data for the longest period available, and in general, this period included the year the surveillance programme started until year 2015 or last year of the surveillance (Figure 1). We used data from 24 ICBDSR member registries or programmes, representing 18 countries in Asia, Europe, North America, and South America. Programmes were eligible to participate in the study if they collected data on both spina bifida prevalence and mortality among infants born with spina bifida. For each programme in our study, we examined indicators describing the type of registry (population‐based vs. hospital‐based systems), coverage, ascertainment period, stillbirth definition, ETOPFA allowed and availability of prenatal screening services (Table 1). Each programme has local procedures for ethics approval, and because this study was done using aggregated data, no additional ethics committee approval was required.

**Table 1:** Description of birth defects registries included in the spina bifida mortality study from registries contributing to the International Clearinghouse for Birth Defects Surveillance and Research

Country‐registry	Type of registry	Coverage	Ascertainment period	Stillbirth definition	ETOPFA allowed	Prenatal screening services
Argentina‐RENAC	H	N	Hospital discharge	>500 g	No	Yes, but no official programme
Colombia‐Bogotá	H	R	1st day	>500 g	Yes, since 2006	Yes
Colombia‐Cali	H	R	1st day	>500 g	Yes, since 2006	Yes
South America‐ECLAMC	H	R^a^	Hospital discharge	>500 g	No^e^	Yes
Czech Republic	P	N	15 y	22 wks or >500 g	Yes	Yes
France‐Paris	P	R	28 d	22 wks	Yes	Yes
Germany‐Saxony Anhalt	P	R	1 y	>500 g	Yes	Yes, since 1990
Italy‐Lombardy	P	R	6 y	23 wks	Yes	Yes
Italy‐Tuscany	P	R	1 y	20 wks	Yes	yes
Malta‐MCAR	P	N	1 y	22 wks	No	Yes, gradually introduced
Netherlands‐Northern	P	R	10 y	24 wks	Yes	Yes, since 2007
Slovak Republic	P	N	Hospital discharge	>500 g	Yes	Yes
Spain‐ECEMC	H	R^b^	3 d	24 wks or 500 g^d^	Yes, since 1985	Yes
Sweden	P	N	Before 1987 1 mo, After 1987 1 y	until 2006:28 wks, 2007 and after: 22 wks	Yes, registration since 1999	Yes, since the early 1980s
UK‐Wales	P	R	18 y	24 wks	Yes	Yes, since 2003
Ukraine‐OMNI‐Net	P	R	1 y	until 2006:28 wks/>1000 g 2006 and after: 22 wks/>500 g	Yes	Yes
Mexico‐Nuevo León	P	R	6 d	Not included	No	Yes, only US
Mexico‐RYVEMCE	H	R	3 d	>=20 gestational weeks or >=500 g	No	No
USA‐Arkansas	P	S	2 y	20 wks	Yes, until 20 wks	Yes
USA‐Atlanta	P	R	6 y	20 wks	Yes	Yes
USA‐Texas	P	S	1 y	20 wks	Yes	Yes
USA‐Utah	P	S	2 y	20 wks	Yes	Yes
Iran‐TROCA	H	R	1 y	20 wks	Yes, restrictions since 2013	Yes
Israel‐SMC	H	R^c^	Hospital discharge	Not included	Yes, but not registered	Yes

Note

Column‐type of programme: H = hospital‐based, P = population‐based; column‐coverage: N = national, R = regional, S = statewide.

Abbreviations: ECEMC, Registry of the Spanish Collaborative Study of Congenital Malformations; ECLAMC, Latin American Collaborative Study of Congenital Malformations; ETOPFA, Elective Termination of Pregnancy for Fetal Anomalies; MCAR, Malta Congenital Anomalies Registry; OMNI‐Net, Ukraine Birth Defects Prevention Program; RENAC, National Network of Congenital Anomalies of Argentina; RYVEMCE, Mexican Registry and Epidemiological Surveillance of External Congenital Malformations; SMC, Soroka Medical Center; TROCA, Tabriz Registry of Congenital Anomalies; UK, United Kingdom; USA, United States of America.

a Several regions in South America.

b Several regions in Spain currently covering around 18% of total births.

c Referral area of one hospital.

d Column‐stillbirth definition: if gestational age of death is not determined (since 1980).

e Column‐ETOPFA allowed except for anencephaly.

### 2.2 Spina bifida—Case definition

International Clearinghouse for Birth Defects Surveillance and Research defined spina bifida as ‘a family of congenital malformation defects in the closure of the spinal column characterized by herniation or exposure of the spinal cord and/or meninges through an incompletely closed spine. Includes: meningocele, meningomyelocele, myelocele, myelomeningocele, and rachischisis. Spina bifida is not counted when present with anencephaly. Excludes: spina bifida occulta, sacrococcygeal teratoma without dysraphism’. This case definition corresponds to *International Classification of Disease* (ICD)‐10 code ‘Q05’ and ICD‐9 code ‘741’. Individual ICBDSR programmes provided information on the annual number of cases with spina bifida and the pregnancy outcome (livebirth, stillbirth, ETOPFA). Spina bifida cases were further classified based on clinical presentation (isolated, multiple congenital anomalies [MCA], syndromic). Isolated cases were defined as those with spina bifida, but with no other co‐occurring unrelated major birth defects. The definition of MCA included spina bifida co‐occurring with one or more unrelated major anomalies. A case was defined as ‘syndromic’ when the spina bifida was part of a genetic disorder or a recognised syndrome.

### 2.3 Mortality

Information on mortality was based on several follow‐up methods as applied by the surveillance programmes, including follow‐up until discharge from the hospital after birth, active or passive follow‐up of the children by clinicians or registry staff, or follow‐up by linking to administrative databases such as death records or other health care databases. Programmes could use more than one follow‐up method. In Table 2, we present the method of follow‐up that was applied in each programme. In our analysis, we examined mortality at different ages, including up to the first day of life, day 2‐6 (early neonatal), day 7‐27 (late neonatal), day 28‐1 year (infant), 1‐4 years (under five), and ≥5 years of age.

**Table 2:** Description of follow‐up method for livebirths by registry from registries contributing to the International Clearinghouse for Birth Defects Surveillance and Research

Country‐registry	Follow‐up until discharge from the maternity hospital	Follow‐up by a clinician or registry staff	Linkage with death certificates	Maximum follow‐up period reported in study
Argentina‐RENAC	Yes	Yes	No	1‐6 d
Colombia‐Bogotá	Yes	Yes	No	1 d
Colombia‐Cali	Yes	Yes	No	No mortality reported for live births
South America‐ECLAMC	Yes	Yes	No	28 d‐11 mo
Czech Republic	No	No	Yes	≥5 y
France‐Paris	Yes	Yes	No	7‐27 d
Germany‐Saxony Anhalt	Yes	Yes^b^	No	1‐4 y
Italy‐Lombardy	No	No	Yes, 2003 up to 2015	1‐4 y
Italy‐Tuscany	No	No	Yes, 1992 up to 2015	28 d‐11 mo
Malta‐MCAR	Yes^a^	Yes	Yes^d^	1‐4 y
Netherlands‐Northern	Yes	Yes	No	≥5 y
Slovak Republic	Yes	No	No	1‐6 d
Spain‐ECEMC	Yes	No	No	1‐6 d
Sweden	No	No	Yes, 1974 up to April 2016	≥5 y
UK‐Wales	Yes	No	Yes, to GP system, till 18 y	≥5 y
Ukraine‐OMNI‐Net	Yes	Yes	No	1‐4 y
Mexico‐Nuevo León	Yes	No	No	1‐6 d
Mexico‐RYVEMCE	Yes	No	No	1‐6 d
USA‐Arkansas	Yes	No	Yes, 1993 up to 2015	≥5 y
USA‐Atlanta	Yes	No	Yes, 1979 up to 2008	≥5 y
USA‐Texas	Yes	No	Yes, 1996 up to 2013	≥5 y
USA‐Utah	Yes	No	Yes, until age 2 y	≥5 y
Iran‐TROCA	Yes	Yes^c^	No	1‐6 d
Israel‐SMC	Yes	No	Yes, 2000 up to 2014	1‐4 y

Abbreviations: ECEMC, Registry of the Spanish Collaborative Study of Congenital Malformations; ECLAMC, Latin American Collaborative Study of Congenital Malformations; GP, General Practitioner; MCAR, Malta Congenital Anomalies Registry; OMNI‐Net, Ukraine Birth Defects Prevention Program; RENAC, National Network of Congenital Anomalies of Argentina; RYVEMCE, Mexican Registry and Epidemiological Surveillance of External Congenital Malformations; SMC, Soroka Medical Center; TROCA, Tabriz Registry of Congenital Anomalies; UK, United Kingdom; USA = United States of America.

Follow‐up method for livebirths:

a Babies are followed up until discharge, and their hospital files are again seen at 1 y of age, linkage with mortality data continues indefinitely.

b Until 18 years.

c Children in university hospital(s).

d Continuous linkage with mortality register, for this study, data have linkage up to 2015.

### 2.4 Statistical analysis

We report results primarily per individual programme, and not as a pooled analysis, because some programmes contributed considerably more cases than others, and because our main goal was to examine variations across individual programmes and countries. For each programme, we calculated total prevalence of spina bifida as the total number of cases with spina bifida (livebirths + stillbirths +ETOPFA for spina bifida) divided by the total number of births (livebirths + stillbirths) in a specified time period. We estimated prevalence and 95% confidence interval for the prevalence estimate using the Poisson approximation of binomial distribution. We did not include ETOPFA in the denominator of the prevalence formula because of lack of data on the total number of terminations for each programme. We estimated the proportion of spina bifida‐affected pregnancies resulting in livebirths, stillbirths, and ETOPFA.

Among liveborn spina bifida cases, we calculated age‐specific mortality as number of deaths among liveborn cases divided by total number of liveborn cases with spina bifida, at different ages (day of birth, days 2‐6, days 7‐27, days 28‐1 year, 1‐4 years, and 5 years or greater), depending on programmes' availability (Table 2). We examined long‐term mortality outcomes in a subset of programmes where linkages to death registration systems (death certificates) or other administrative databases allowed a lengthy follow‐up beyond the neonatal period. Survival proportion was calculated in each programme by extracting the cumulative proportion of cases who died at ages specified above from the total number of livebirths with spina bifida (set at 100% at birth). For programmes where data were available, we examined mortality by isolated and non‐isolated case status (combining MCA and syndromic cases).

Finally, we examined trends in ETOPFA, stillbirth, and first‐week mortality for each programme for the total available study period. Because of small numbers, we calculated the proportion of spina bifida‐affected pregnancies resulting in ETOPFA, stillbirth, and first‐week mortality in livebirths by pooling 5‐year periods, starting from 1976 (or first year available) to 2014 (or last year available). We used 5‐year periods (1976‐1980, 1981‐1985, and so on); for some programmes, the first and last period may be fewer than 5 years. We did not report results by single years or for programmes that had fewer than 5 cases.

## 3 RESULTS

### 3.1 Programme characteristics

A total of 24 ICBDSR member programmes with birth defects registries representing 18 countries contributed data for a part or entire time period between years 1974 and 2015 examined in our analysis (Figure 1). Sixteen of the 24 registries were population‐based, with regional (n = 10), statewide (n = 3), or national (n = 3) coverage. The maximum age of ascertainment for birth defects varied by programme; however, most cases of spina bifida can be easily identified at birth. Criteria to define stillbirths also varied. ETOPFA was not allowed in the surveillance region for 5 of the 24 programmes. In all regions covered by the ICBDSR programmes included in our analysis, prenatal screening services were offered in recent years (Table 1).

Mortality analysis was mostly restricted to a short postnatal follow‐up (Table 2). Postnatal follow‐up was performed from birth until discharge from the birth hospital in 20 out of the 24 participating programmes. The four programmes that did not collect information on vital status at hospital discharge or during the delivery hospitalisation (Czech Republic, Italy‐Lombardy, Italy‐Tuscany, and Sweden) used linkage to vital records to collect information on vital status. In total, there were 11 programmes that used linkages to death certificates or other health care databases to determine vital status (Czech Republic, Italy‐Lombardy, Italy‐Tuscany, Malta‐MCAR, Sweden, UK‐Wales, USA‐Arkansas, USA‐Atlanta, USA‐Texas, USA‐Utah, and Israel‐Soroka Medical Center). Three programmes collected only information on vital status at hospital discharge and did not use other follow‐up methods (Slovak Republic, Mexico‐Nuevo Leon, and Mexico‐RYVEMCE). Maximum follow‐up period noting infant survival varied by programme, but all programmes provided information on mortality that occurred in the first week of life.

### 3.2 Prevalence and pregnancy outcome

Since most programmes provided data for surveillance years 2001‐2012 (83% of programmes presented data on 2001, increasing to more than 95% of programmes in 2012), we elected to focus our analysis on this time interval. This allowed comparison of results between programmes. In Table 3, we present programme‐specific spina bifida prevalence per 10 000 total births and the pregnancy outcome. The prevalence of spina bifida during 2001‐2012 was 4.7 per 10 000 total births (95% CI 4.6, 4.8). The highest prevalence estimates of spina bifida per 10 000 total births were observed in Ukraine‐OMNI‐Net (10.9), South America‐ECLAMC (9.9), and UK‐Wales (7.3), whereas Mexico‐Nuevo León (1.4), Iran (1.6), Colombia‐Cali (2.4), and Czech Republic (2.9) showed the lowest prevalence. The highest proportion of ETOPFA among spina bifida cases were observed in four European registries with over 70% of pregnancies affected with spina bifida electively terminated (Spain‐ECEMC, 84.6%; France‐Paris, 81.0%; Italy‐Tuscany, 76.5%; and UK‐Wales, 72.7%). Overall, the proportion of stillbirths ranged between 0% and 10%, and the proportion of stillbirths was highest in programmes from countries that do not allow termination of pregnancy.

**Table 3:** Total number of births, total number of spina bifida cases and prevalence per 10 000 births, proportion of livebirth among total cases of spina bifida, proportion of stillbirths among total cases of spina bifida, and proportion of ETOPFA among total cases of spina bifida for surveillance period 2001‐2012 from registries contributing to the International Clearinghouse for Birth Defects Surveillance and Research

Country‐registry	Type of registry	Surveillance period	Total births	Total cases of spina bifida	Total prevalence per 10 000 total births (95% CI)	Livebirth % (95% CI)	Stillbirth % (95% CI)	ETOPFA % (95% CI)
Argentina‐RENAC^a^	H	2009‐2012	422 173	241	5.7 (5.0, 6.5)	94.6 (91.0, 96.8)	5.0 (2.9, 8.5)	–
Colombia‐Bogotá^b^	H	2001‐2012	356 454	113	3.2 (2.6, 3.8)	92.0 (85.6, 95.6)	8.0 (4.2, 14.4)	–
Colombia‐Cali^b^	H	2011‐2012	12 762	3	2.4 (0.5, 6.9)	100 (43.9, 100)	0 (0, 56.2)	–
South America‐ECLAMC^a^	H	2001‐2012	1 847 181	1819	9.9 (9.4, 10.3)	93.5 (92.2, 94.5)	6.5 (5.5, 7.8)	–
Czech Republic	P	2001‐2012	1 273 386	367	2.9 (2.6, 3.2)	37.9 (33.1, 42.9)	1.4 (0.6, 3.2)	60.8 (55.7,65.6)
France‐Paris	P	2001‐2012	319 636	184	5.8 (5.0, 6.7)	18.5 (13.2, 24.7)	0.5 (0.1, 3.0)	81.0 (74.7, 86.0)
Germany‐Saxony Anhalt	P	2001‐2012	208 108	121	5.8 (4.8, 7.0)	33.8 (26.1, 42.7)	1.7 (0.5, 5.8)	64.5 (55.6, 72.4)
Italy‐Lombardy	P	2003‐2012	133 182	64	4.8 (3.7, 6.1)	39.1 (28.1, 51.3)	1.6 (0.3, 8.3)	59.4 (47.2, 70.5)
Italy‐Tuscany	P	2001‐2012	352 844	108	3.1 (2.5, 3.7)	20.4 (13.9, 28.9)	2.8 (1.0, 7.9)	76.5 (68.1, 83.8)
Malta‐MCAR^a^	P	2001‐2012	48 202	31	6.4 (4.4, 9.1)	90.3 (75.1, 96.7)	9.7 (3.3, 24.9)	–
Netherlands‐Northern	P	2001‐2012	221 846	106	4.8 (3.9, 5.8)	53.8 (44.3, 63.0)	7.5 (3.9, 14.2)	38.7 (30.0, 48.2)
Slovak Republic	P	2001‐2012	667 992	224	3.4 (2.9, 3.8)	79.9 (74.2, 84.6)	2.2 (1.0, 5.1)	17.9 (13.4, 23.4)
Spain‐ECEMC	H	2001‐2012	259 285	156	6.0 (5.1, 7.0)	14.7 (10.0, 21.2)	0.6 (0.1, 3.5)	84.6 (78.1, 89.4)
Sweden	P	2001‐2012	1 230 002	583	4.7 (4.4, 5.1)	45.1 (41.1, 49.2)	0.3 (0.1, 1.2)	54.5 (50.5, 58.5)
Ukraine‐OMNI‐Net	P	2001‐2012	347 418	378	10.9 (9.8, 12.0)	44.7 (39.8, 49.8)	3.2 (1.8, 5.5)	47.4 (42.4, 52.4)
UK‐Wales	P	2001‐2012	404 385	297	7.3 (6.5, 8.2)	26.3 (21.6, 31.6)	1.0 (0.3, 2.9)	72.7 (67.4, 77.5)
Mexico‐Nuevo León^a^	P	2011‐2012	168 661	23	1.4 (0.9, 2.1)	100 (85.7, 100)	0 (0, 14.3)	–
Mexico‐RYVEMCE^a^	H	2001‐2012	264 306	169	6.4 (5.5, 7.4)	94.7 (90.2, 97.2)	5.3 (2.8, 9.8)	–
USA‐Arkansas	P	2001‐2012	470 593	207	4.4 (3.8, 5.0)	85.5 (80.1, 89.7)	4.8 (2.6, 8.7)	7.2 (4.4, 11.6)
USA‐Atlanta	P	2001‐2008	428 976	180	4.2 (3.6, 4.9)	62.2 (55.0, 69.0)	8.9 (5.5, 14.0)	25.0 (19.2, 31.8)
USA‐Texas	P	2001‐2012	4 668 071	1737	3.7 (3.6, 3.9)	90.8 (86.4, 92.1)	4.0 (3.2, 5.1)	5.1 (4.2, 6.3)
USA‐Utah	P	2001‐2012	624 990	249	4.0 (3.5, 4.5)	85.5 (80.6, 89.4)	4.4 (2.5, 7.7)	10.0 (6.9, 14.4)
Iran‐TROCA	H	2004‐2012	160 755	25	1.6 (1.0, 2.3)	88.0 (70.0, 95.8)	4.0 (0.7, 19.5)	8 (2.2, 24.9)
Israel‐SMC^c^	H	2001‐2012	158 544	47	3.0 (2.2, 3.9)	100 (92.4, 100)	0 (0, 7.6)	–
Total		2001‐2012	15 049 752	7432	4.7 (4.6, 4.8)	73.2 (72.2, 74.3)	4.1 (3.7, 4.6)	33.6 (32.0, 35.2)^d^

Abbreviations: CI, confidence Interval; ECEMC, Registry of the Spanish Collaborative Study of Congenital Malformations; ECLAMC, Latin American Collaborative Study of Congenital Malformations; ETOPFA, Elective Termination of Pregnancy for Fetal Anomalies; H, hospital‐based programme; MCAR, Malta Congenital Anomalies Registry; OMNI‐Net, Ukraine Birth Defects Prevention Program; P, population‐based programme; RENAC, National Network of Congenital Anomalies of Argentina; RYVEMCE, Mexican Registry and Epidemiological Surveillance of External Congenital Malformations; SMC, Soroka Medical Center; TROCA, Tabriz Registry of Congenital Anomalies; UK, United Kingdom; USA, United States of America.

a ETOPFA not allowed.

b ETOPFA not registered.

c Data on liveborn children with spina bifida from one hospital.

d Excludes programmes where ETOPFA is unavailable, or does not report on ETOPFA.

The overall results on prevalence of spina bifida, examined as a secondary objective of the study, for the complete surveillance period (1974‐2015), are presented in Table S1. The total number of births covered by all programmes was 28 213 327 (including livebirths and stillbirths), and the total number of spina bifida cases equalled to 14 159. Thus, the prevalence of spina bifida in our study was estimated to be 5.0 per 10 000 total births (95% CI 4.9, 5.1).

### 3.3 Mortality

The overall first‐week mortality (years 2001‐2012) proportion was 6.9% (95% CI 6.3%, 7.7%) (Table 4). The first‐week mortality proportion was lower in programmes where ETOPFA was available. When taking isolated and complex cases together, the majority of deaths occurring during the first week of life were reported to have been during the first day of life. However, in Malta (based on 1 case), Northern Netherlands, and Israel, the majority of spina bifida deaths occurred after the first day. Most (~80%) deaths occurred within the first 28 days of life (neonatal period).

**Table 4:** Mortality in spina bifida‐affected births for surveillance period 2001‐2012 from registries contributing to the International Clearinghouse for Birth Defects Surveillance and Research

Country‐registry	Surveillance period	Livebirths with spina bifida	Age at death^f^					
			Day 1	Day 2‐Day 6	Day 7‐Day 27	Day 28‐Month 12	Year 1‐4	Year 5 and above
		N	%	%	%	%	%	%
Argentina‐RENAC^a^	2009‐2012	228	9.6^d^		–	–	–	–
Colombia‐Bogotá^b^	2001‐2012	104	4.8	–	–	–	–	–
South America‐ECLAMC^a^	2001‐2012	1700	6.5	2.5	1.6	0.8	–	–
Czech Republic	2001‐2012	139	0.7	2.9	3.6	1.4	1.4	1.4
France‐Paris^b^	2001‐2012	34	2.9	2.9	2.9		–	–
Germany‐Saxony Anhalt	2001‐2012	41	0	0	0	0	0	–
Italy‐Lombardy	2003‐2012	25	0	0	0	0	4.0	–
Italy‐Tuscany	2001‐2012	22	4.5	0	0	0	–	–
Malta^a^	2001‐2012	28	0.0	3.6	3.6	0	0	–
Netherlands‐Northern	2001‐2012	55	7.3	18.2	12.7	0	0	0
Slovak Republic	2001‐2012	179	0	5.0	–	–	–	–
Spain‐ECEMC	2001‐2012	23	8.7	0	–	–	–	0
Sweden	2001‐2012	263	1.5	2.7	1.9	1.5	0.4	0.4
Ukraine‐OMNI‐Net	2001‐2012	169	3.0	2.4	1.8	14.8	1.8	–
UK‐Wales	2001‐2012	78	6.4	1.3	1.3	0	1.3	0
Mexico‐Nuevo León^a^	2011‐2012	23	0	0	–	–	–	–
Mexico‐RYVEMCE^a^	2001‐2012	160	3.1	1.3	–	–	–	–
USA‐Arkansas	2001‐2012	177	6.2	2.3	1.1	3.4	2.8	1.1
USA‐Atlanta	2001‐2008	112	0.9	0.9	1.8	0.9	0	0
USA‐Texas	2001‐2012	1578	3.5	1.7	1.0	2.2	1.1	0.4
USA‐Utah	2001‐2012	213	6.1	1.4	0.9	0.9	0	0.5
Iran‐TROCA	2004‐2012	22	0.0	4.5	–	–	–	–
Israel‐Soroka Medical Center^c^	2001‐2012	47	6.4	14.9	14.9	4.3	2.1	–
Total		5420	4.2	2.7	1.7^e^	1.9^e^	1.1^e^	0.4^e^

Abbreviations: ECEMC, Registry of the Spanish Collaborative Study of Congenital Malformations; ECLAMC, Latin American Collaborative Study of Congenital Malformations; LB, livebirth; MCAR, Malta Congenital Anomalies Registry; OMNI‐Net, Ukraine Birth Defects Prevention Program; RENAC, National Network of Congenital Anomalies of Argentina; RYVEMCE, Mexican Registry and Epidemiological Surveillance of External Congenital Malformations; SB, Stillbirth; SMC, Soroka Medical Center; TROCA, Tabriz Registry of Congenital Anomalies; UK, United Kingdom; USA, United States of America.

a ETOPFA not allowed.

b ETOPFA not registered.

c Data on liveborn children with spina bifida from one hospital.

d Percentage refers to first‐week mortality.

e Excludes programmes that have no data on mortality for selected age at death.

f A hyphen means that the registry did not report follow‐up data for that time period.

A total of 17 programmes provided data on additional birth defects co‐occurring with spina bifida. Since the number of spina bifida cases with a syndromic aetiology was very small, we pooled them with the MCA cases in the analyses. On average, 68% presented as isolated and 32% as MCA or as part of a genetic syndrome (Table 5). The distribution varied by programme; in Israel and Mexico‐RYVEMCE, more than 90% of spina bifida cases were described as isolated, whereas in Italy‐Lombardy and in South America‐ECLAMC, 53% and 51% were described as isolated, respectively. In Europe, where ETOPFA is allowed and common, ETOPFA proportion was similar between isolated and MCA/syndromic cases. Among all programmes, the proportion of first‐day and first‐week mortality was higher in MCA/syndromic cases compared with isolated cases (Table 5).

**Table 5:** Type of birth and first‐week mortality among livebirths affected with spina bifida according to clinical presentation from registries contributing to the International Clearinghouse for Birth Defects Surveillance and Research, 2001‐2012

Country‐registry	Isolated spina bifida							Multiple/syndromic spina bifida						
	Total cases		Type of Birth			Mortality in LB		Total cases		Type of Birth			Mortality in LB	
			ETOPFA	SB	LB	Day 1	Day 2‐6			ETOPFA	SB	LB	Day 1	Day 2‐6
	N	%	%	%	%	%	%	N	%	%	%	%	%	%
Argentina‐RENAC^a^	189	78	–	2	98	3		52	22	–	17	83	0	40
Colombia‐Bogotá^b^	96	85	–	5	94	2	0	17	15	–	24	76	15	0
SA‐ECLAMC^a^	935	51	–	3	97	2	1	884	49	–	10	90	12	4
France‐Paris	139	76	81	1	18	0	4	45	24	80	0	20	11	0
Germany‐Saxony Anhalt	103	85	65	1	34	0	0	18	15	61	6	33	0	0
Italy‐Lombardy	34	51	59	0	41	0	0	33	49	64	3	33	0	0
Italy‐Tuscany	73	68	77	0	23	0	0	35	32	77	9	14	20	0
Malta‐MCAR^a^	22	71	–	0	100	0	5	9	29	–	33	67	0	0
Netherlands‐Northern	80	75	39	4	58	2	17	26	25	46	19	35	33	22
Slovak Republic	150	67	15	1	84	0	1	74	33	24	4	72	0	15
Spain‐ECEMC	112	72	83	0	17	5	0	44	28	89	2	9	25	0
Sweden	441	76	58	0	42	1	2	142	24	44	1	56	3	5
Ukraine‐OMNI‐Net^e^	333	88	48	3	44	1	1	45	12	42	4	49	18	14
UK‐Wales	238	82	75	0	25	3	0	54	18	63	4	33	17	6
Mexico‐RYVEMCE^a^	153	91	–	3	97	2	1	16	9	–	31	69	9	0
USA‐Utah	174	70	11	2	87	2	1	75	30	8	11	81	16	2
Israel‐SMC^c^	45	96	–	–	100	2	16	2	4	–	–	100	100	0
Total	3317	68	54^d^	2	67	1	2	1571	32	48^d^	9	73	11	6

Note

Frequencies for mortality statistics are not presented due to sparse data.

Abbreviations: ECEMC, Registry of the Spanish Collaborative Study of Congenital Malformations; ECLAMC, Latin American Collaborative Study of Congenital Malformations; ETOPFA, Elective Termination of Pregnancy for Fetal Anomalies; LB, livebirth; MCAR, Malta Congenital Anomalies Registry; OMNI‐Net, Ukraine Birth Defects Prevention Program; RENAC, National Network of Congenital Anomalies of Argentina; RYVEMCE, Mexican Registry and Epidemiological Surveillance of External Congenital Malformations; SB, Stillbirth; SMC, Soroka Medical Center; TROCA, Tabriz Registry of Congenital Anomalies; UK, United Kingdom; USA, United States of America.

a ETOPFA not allowed.

b ETOPFA not registered.

c Data on liveborn children with spina bifida from one hospital.

d Excludes programmes where ETOPFA is unavailable, or does not report on ETOPFA.

e Type of birth unknown for 16 isolated cases (5%) and 1 multiple/syndromic case.

**Figure 2:**
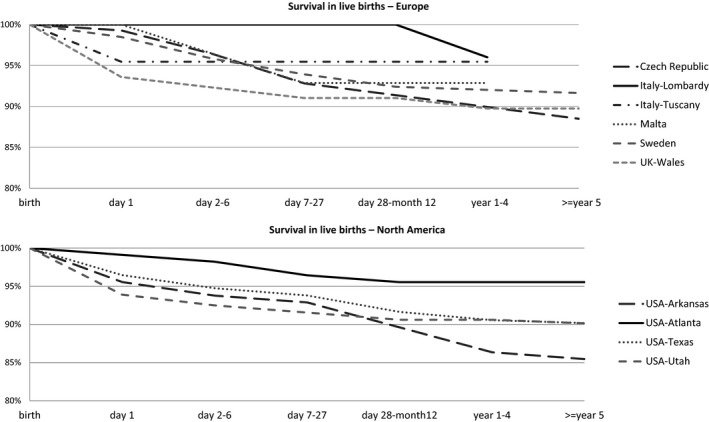
Survival in livebirths with spina bifida for surveillance period 2001‐2012, from European and North American registries (with linkage to administrative databases) contributing to the International Clearinghouse for Birth Defects Surveillance and Research

Only 10 programmes, from Europe and North America, provided information on long‐term mortality (over age 1 year) using linkage to vital records. These programmes were Czech Republic, Italy‐Lombardy, Malta‐MCAR, Sweden, Italy‐Tuscany, UK‐Wales, USA‐Arkansas, USA‐Atlanta, USA‐Texas, and USA‐Utah. In Europe, survival up to age 1‐4 years was 90%‐96%, and in North American programmes, the survival was similar, at 86%‐96% (Figure 2).

Trends in ETOPFA, stillbirths, and first‐week mortality in livebirths affected with spina bifida from registries contributing to the ICBDSR are presented in Figure S1. Looking at pooled 5‐year, programme‐specific trends in mortality, we found that in most European programmes an increase in ETOPFA proportion was observed over time, which was accompanied by a decrease in stillbirths and first‐week mortality (eg Czech Republic, France‐Paris, Germany‐Saxony Anhalt, Italy, Northern Netherlands, Spain, and Sweden). In the Slovak Republic, the proportion of ETOPFA, stillbirth, and first‐week mortality declined in the most recent study period (2011‐2014). In UK‐Wales, a decline in ETOPFA was also observed but with stable stillbirth and first‐week mortality proportions. In the United States, a decreasing trend in the ETOPFA proportion was observed during the same period, with a relatively stable or decreasing stillbirth and first‐week mortality proportions (except for USA‐Texas, where an increase in first‐week mortality was observed). In Mexico‐RYVMCE, a decreasing trend in both the ETOPFA proportion and the stillbirth proportions were observed.

## 4 COMMENT

### 4.1 Principal findings

This is the first multi‐country, multi‐registry study that provides estimates of prevalence, perinatal and infant and child mortality, and mortality trends among those born with spina bifida using data from 24 birth defects programmes in 18 countries affiliated with the ICBDSR. Our findings confirm that a substantial proportion of pregnancies affected by spina bifida end in ETOPFA, stillbirths, or infant mortality in the countries examined. The highest ETOPFA proportion was observed in some European registries, with over 70% of pregnancies affected with spina bifida electively terminated. Overall, up to 10% of infants born with spina bifida died either on the first day or by the first month of life. We observed a higher occurrence of stillbirths and neonatal mortality in countries in which termination of pregnancy after fetal diagnosis for congenital malformations was not available. We also found that the proportion of perinatal deaths were higher among cases with other anomalies or genetic syndromes compared with isolated cases. Our study allowed a comparison of findings between participating programmes.

### 4.2 Strengths of the study

Our study was the first to examine perinatal and infant child mortality among those born with spina bifida in a diverse set of populations using multi‐registry, multi‐country data. International Clearinghouse for Birth Defects Surveillance and Research programmes have quality control protocols to enhance case specificity for spina bifida, while tracking cases from multiple data sources. We were able to examine all birth outcomes, including stillbirths and ETOPFA. Information on whether infants with spina bifida had additional birth defects was available for most programmes. Mortality outcomes were examined by age. Most programmes had information on mortality during the first week of life, and we could compare findings between programmes. Programmes provided information on existing policies on ETOPFA, which allowed us to compare findings by ETOPFA policies. Mortality outcomes were pooled by isolated and non‐isolated cases of spina bifida.

### 4.3 Limitations of the data

There were several limitations in our study. First, programmes that contributed data were not homogenous in methods; and the surveillance periods varied. We did not have individual‐level data. We may have missed some cases as programmes may not have captured all stillbirths and ETOPFA cases. Accuracy of mortality outcomes, and age at death, cannot be confirmed. Data linkages with death certificates were not uniform across all programmes. We were unable to examine temporal trends in mortality for programmes that provided data for short durations (ie <5 years). There may have been deaths that could not be tracked due to limitations in administrative data linkages, or if they occurred outside the programme surveillance area.

### 4.4 Interpretation

The average prevalence of spina bifida (including all cases) in our analysis, using data from 24 programmes reporting to ICBDSR, was 5.0 per 10 000 total births. This prevalence is very similar to the total prevalence reported by programmes that are full members of the EUROCAT (European Surveillance of Congenital Anomalies) network, which includes most population‐based congenital anomaly registries in Europe with a common database (http://www.eurocat-network.eu/), at 5.1 per 10 000 total births, between years 1980 and 2015. Some of the surveillance programmes participate in both ICBDSR and EUROCAT (full members) (France‐Paris, Germany‐Saxony Anhalt, Italy‐Tuscany, Malta‐MCAR, Northern Netherlands, Ukraine‐OMNI‐Net, and UK‐Wales). Our analysis included countries outside the EUROCAT network, including Argentina, Colombia, South America‐ECLAMC, Czech Republic, Mexico, USA, Iran, and Israel, contributing additional data to the existing literature from EUROCAT countries.

We noted that the first‐week mortality among spina bifida cases was highest in Northern Netherlands (25.5%) and Israel (21.3%). In the Northern Netherlands, a prenatal screening programme was introduced in 2007, after which the first‐week mortality dropped from 31.4% to 15.0% (data not shown). The data from Israel are from one hospital and therefore may not be representative of the country.

Survival among spina bifida cases is known to be influenced negatively by low birthweight and high lesions, and positively by surgical interventions soon after birth.^20^, ^22^, ^23^ In our study, we also observed that mortality in cases with isolated spina bifida is lower than in cases with spina bifida and other anomalies or syndromes. A probable explanation is that isolated cases are less complicated. Overall, the general consensus is that the survival probability in developed countries is about 80% up to age 1 year,^16^, ^24^ and the higher probability of death persists with increasing age among those with spina bifida compared to those without.^16^, ^17^, ^25^ Wang et al (2010) reported that the relative risk of death among children born with spina bifida is 10 times (95% CI 7.5, 13.5) greater compared with children born without birth defects at age 6 or older.^26^ Sex, age, race and ethnicity, severity of the lesion, multiple birth defects, birth year, and availability, use, and acceptance of medical and surgical treatments have been associated with variations in mortality in spina bifida worldwide.^1^, ^16^, ^17^, ^18^

Population‐based state registry data with linkage to death certificates in New York, USA, identified hydrocephalus, infections, cardiac anomalies, pneumonia, and pulmonary embolism as common causes of death in children with spina bifida.^18^ We were unable to examine specific causes of death and the influence of birthweight, lesion severity, and other aforementioned factors on mortality associated with spina bifida, as data were unavailable and beyond the scope of our current analysis.

We noted an increasing trend in ETOPFA for spina bifida in European programmes, with a concomitant decrease in stillbirths and first‐week mortality proportions. A retrospective cohort study from the Netherlands examining the impact of introduction of the mid‐trimester scan during the year 2007 on pregnancy outcome of spina bifida cases diagnosed pre‐ or postnatally reported that pregnancies that previously might have ended in a perinatal loss are now terminated, while pregnancies with a relatively good prognosis are frequently not terminated; the overall number of liveborn children with spina bifida has not changed significantly.^27^

## 5 CONCLUSIONS

Data from 24 programmes provided a first summary of spina bifida‐associated perinatal and infant and child mortality and their trends. In the many countries that contributed data for our analysis, mortality among those affected with spina bifida is a major concern, especially during the first day and first week of life. Additional data, including sociodemographic and clinical factors, could be utilised to further understand disparities in mortality that we observed in different programmes. Mortality in spina bifida is preventable through timely surgical and medical care at birth and beyond, and advanced health care throughout the life course of those affected. Findings from our analysis can inform policymakers of the need for primary prevention of spina bifida with folic acid interventions to address preventable mortality associated with this severe and often fatal birth defect. Primary prevention of spina bifida through folic acid fortification and supplementation should be considered the first policy in any country to avoid unnecessary disability and mortality associated with spina bifida.
