# Supplementary figures and images for: Analysis of Mortality among Neonates and Children with Spina Bifida: An International Registry‐Based Study, 2001‐2012
Source: Paediatr Perinat Epidemiol. 2019 Oct 21;33(6):436–48. doi: 10.1111/ppe.12589 (PMC6899817; doi:10.1111/ppe.12589)

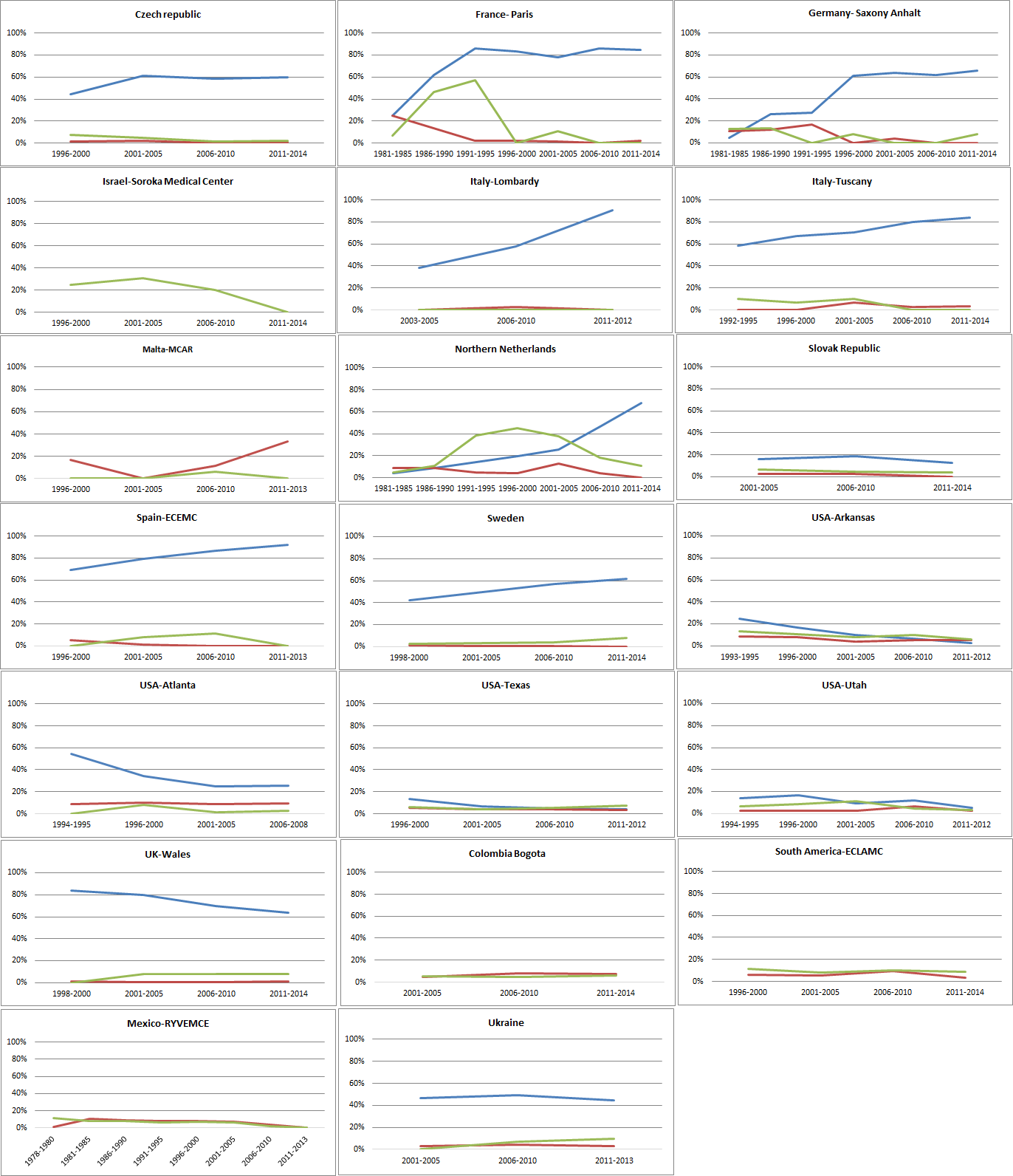

Supplement: Supplementary file 1 [file PPE-33-436-s001.tif]
