# Supplementary material for: Analysis of Mortality among Neonates and Children with Spina Bifida: An International Registry‐Based Study, 2001‐2012
Source: Paediatr Perinat Epidemiol. 2019 Oct 21;33(6):436–48. doi: 10.1111/ppe.12589 (PMC6899817; doi:10.1111/ppe.12589)
Supplement: Supplementary file 2 [file PPE-33-436-s002.docx]

**eTable 1.** Registries and birth years, total number of births and total number of cases, total prevalence, live birth proportion, ETOPFA proportion and first week mortality among live birth proportion for the total surveillance period, International Clearinghouse for Birth Defects Surveillance and Research.

| **Country-Registry** | **Surveillance Period** | **Total Births**  **n** | **Total Spina Bifida**  **n** | **Total prevalence per 10,000 births  (95% CI)** | | **ETOPFA**  **%** | **Stillbirth**  **%** | **Live birth**  **%** | **1 week mortality**  **among live births**  **%** |
| --- | --- | --- | --- | --- | --- | --- | --- | --- | --- |
| Argentina-RENAC^1^ | 2009-2014 | 1,023,108 | 548 | 5.4 | (4.9, 5.8) | - | 4.0% | 95.8% | 11.8% |
| Colombia-Bogotá^2^ | 2000-2014 | 407,394 | 123 | 3.0 | (2.5, 3.6) | - | 7.3% | 92.7% | 5.3% |
| Colombia-Cali^2^ | 2011-2014 | 27,294 | 6 | 2.2 | (1.0, 4.9) | - | 16.7% | 83.3% | nr |
| South America-ECLAMC^1^ | 1995-2014 | 2,927,555 | 2,910 | 9.9 | (9.6, 10.3) | - | 6.1% | 93.9% | 9.6% |
| Czech Republic | 1994-2014 | 2,147,532 | 614 | 2.9 | (2.6, 3.1) | 52.4% | 1.5% | 46.1% | 3.9% |
| France-Paris | 1981-2014 | 875,241 | 440 | 5.0 | (4.6, 5.5) | 72.3% | 6.1% | 21.6% | 15.8% |
| Germany-Saxony Anhalt | 1980-2014 | 526,289 | 324 | 6.2 | (5.5, 6.9) | 42.6% | 6.2% | 51.2% | 8.4% |
| Italy-Lombardy | 2003-2012 | 133,182 | 64 | 4.8 | (3.8, 6.1) | 59.4% | 1.6% | 39.1% | 0.0% |
| Italy-Tuscany | 1992-2014 | 636,562 | 193 | 3.0 | (2.6, 3.5) | 73.1% | 2.1% | 24.9% | 6.3% |
| Malta-MCAR^1^ | 1995-2013 | 79,948 | 52 | 6.5 | (5.0, 8.5) | - | 11.5% | 88.5% | 4.3% |
| Netherlands-Northern | 1981-2014 | 562,462 | 314 | 5.6 | (5.0, 6.2) | 25.2% | 6.4% | 68.5% | 27.0% |
| Slovak Republic | 2001-2014 | 778,177 | 245 | 3.2 | (2.8, 3.6) | 16.3% | 2.0% | 81.6% | 5.5% |
| Spain-ECEMC | 1986-2013 | 2,135,249 | 449 | 2.1 | (1.9, 2.3) | - | 5.1% | 94.9% | 7.3% |
| Spain-ECEMC (Incl. ETOPFA) | 1995-2013 | 373,698 | 245 | 6.6 | (5.8, 7.4) | 80.0% | 2.0% | 18.0% | 6.8% |
| Sweden | 1974-1997 | 2,477,130 | 872 | 3.5 | (3.3, 3.8) | - | 1.7% | 98.3% | 6.8% |
| Sweden (Incl. ETOPFA) | 1998-2014 | 1,718,393 | 807 | 4.7 | (4.4, 5.0) | 53.0% | 0.5% | 46.5% | 4.0% |
| Ukraine | 2000-2013 | 404,172 | 439 | 10.9 | (9.9, 11.9) | 46.9% | 3.9% | 44.6% | 5.1% |
| UK-Wales | 1998-2014 | 569,341 | 431 | 7.6 | (6.9, 8.3) | 73.8% | 0.9% | 25.3% | 7.3% |
| Mexico-RYVEMCE^1^ | 1978-2013 | 1,198,579 | 1,499 | 12.5 | (11.9, 13.2) | - | 7.7% | 92.3% | 7.4% |
| Mexico-Nuevo León^1^ | 2011-2015 | 442,674 | 88 | 2.0 | (1.6, 2.5) | - | 0.0% | 84.1% | 1.4% |
| USA-Atlanta | 1994-2008 | 737,250 | 329 | 4.5 | (4.0, 5.0) | 31.9% | 9.1% | 54.4% | 3.3% |
| USA-Arkansas | 1993-2012 | 760,777 | 701 | 9.2 | (8.6, 9.9) | 8.3% | 3.9% | 86.7% | 7.4% |
| USA-Texas | 1996-2012 | 5,980,798 | 2,304 | 3.9 | (3.7, 4.0) | 7.2% | 4.3% | 88.5% | 5.4% |
| USA-Utah | 1994-2012 | 928,107 | 369 | 4.0 | (3.6, 4.4) | 11.9% | 3.8% | 84.3% | 7.7% |
| Iran-TROCA | 2004-2012 | 160,755 | 25 | 1.6 | (1.1, 2.3) | 8.0% | 4.0% | 88.0% | 4.5% |
| Israel-SMC^3^ | 2000-2014 | 201,660 | 68 | 3.4 | (2.7, 4.3) | - | 0.0% | 100.0% | 17.6% |
| **TOTAL** |  | **28,213,327** | **14,159** | **5.0** | **(4.9, 5.1)** | **34.4%** | **4.6%** | **76.7%** | **7.9%** |

^1^ ETOPFA not allowed; ^2^ ETOPFA not registered; ^3^ data on live born children with spina bifida from one hospital

ECEMC=Registry of the Spanish Collaborative Study of Congenital Malformations; ECLAMC=Latin American Collaborative Study of Congenital Malformations; ETOPFA=Elective termination of pregnancy for fetal anomalies; MCAR=Malta Congenital Anomalies Registry; nr = not reported ; OMNI-Net=Ukraine Birth Defects Prevention Program; RENAC=National Network of Congenital Anomalies of Argentina; RYVEMCE= Mexican Registry and Epidemiological Surveillance of External Congenital Malformations; SB=Stillbirth; TROCA=Tabriz Registry of Congenital Anomalies; SMC=Soroka Medical Center; UK=United Kingdom; USA=United States of America.

**eFigure 1**. Trends in elective termination of pregnancies, stillbirths, and first week mortality in live births affected with spina bifida from registries contributing to the International Clearinghouse for Birth Defects Surveillance and Research.

Blue line = proportion of elective termination of pregnancy for fetal anomalies; Red line = proportion of stillbirths; Green line = proportion of first week mortality in live births. Trends for Argentina-RENAC and Iran-TROCA are not presented due to limited surveillance follow-up data.

ECEMC=Registry of the Spanish Collaborative Study of Congenital Malformations; ECLAMC=Latin American Collaborative Study of Congenital Malformations; MCAR=Malta Congenital Anomalies Registry; OMNI-Net=Ukraine Birth Defects Prevention Program; RENAC=National Network of Congenital Anomalies of Argentina; RYVEMCE= Mexican Registry and Epidemiological Surveillance of External Congenital Malformations; TROCA=Tabriz Registry of Congenital Anomalies; SMC=Soroka Medical Center; UK=United Kingdom; USA=United States of America.
